# Halide-containing organic persistent luminescent materials for environmental sensing applications

**DOI:** 10.1039/d1sc06586f

**Published:** 2021-12-20

**Authors:** Feiyang Li, Mengzhu Wang, Shujuan Liu, Qiang Zhao

**Affiliations:** State Key Laboratory of Organic Electronics and Information Displays & Jiangsu Key Laboratory for Biosensors, Institute of Advanced Materials (IAM) & Institute of Flexible Electronics (Future Technology), Nanjing University of Posts and Telecommunications (NUPT) 9 Wenyuan Road Nanjing 210023 Jiangsu China iamsjliu@njupt.edu.cn iamqzhao@njupt.edu.cn; College of Electronic and Optical Engineering, College of Flexible Electronics (Future Technology), Jiangsu Province Engineering Research Center for Fabrication and Application of Special Optical Fiber Materials and Devices, Nanjing University of Posts and Telecommunications (NUPT) 9 Wenyuan Road Nanjing 210023 Jiangsu China

## Abstract

Great progress has been made in the development of various organic persistent luminescent (OPL) materials in the past few years, and increasing attention has been paid to their interesting applications in environmental sensing due to their long emission lifetimes and high sensitivity. Especially, the introduction of different halogen elements facilitates highly efficient OPL emission with distinct lifetimes and colours. In this review, we summarize the current status of the halide-containing OPL materials for environmental sensing applications. To begin with, the photophysical processes and luminescence mechanisms of OPL materials are expounded in detail to better understand the relationship among molecular structures, OPL properties, and sensing applications. Then, representative halide-containing material systems, such as small molecules, polymers, and doping systems, are summarized with their interesting applications in sensing temperature, oxygen, H_2_O, UV light and organic solvents. In addition, several challenges and future research opportunities in this field are discussed. This review aims to provide some reasonable guidance on the material design of OPL sensors and their practical applications, and tries to provide a new perspective on the application direction of organic optoelectronics.

## Abstract 2

This review presents a summary of the molecular design of halide-containing organic persistent luminescent materials, and their environmental sensing applications.

## Graphical abstract

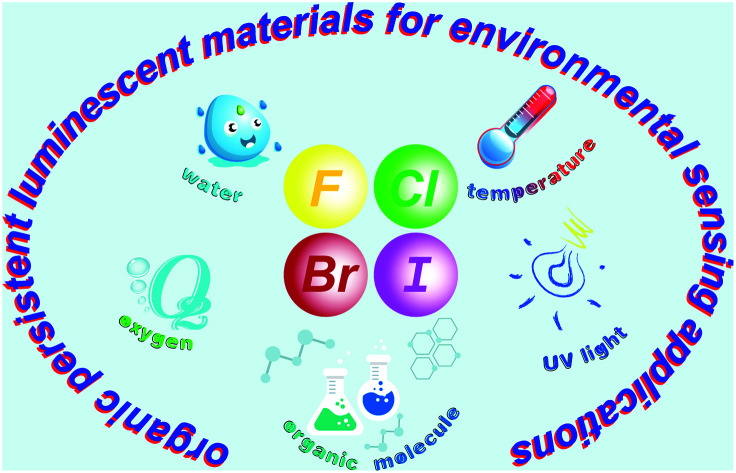

## 1. Introduction

**Scheme 1:**
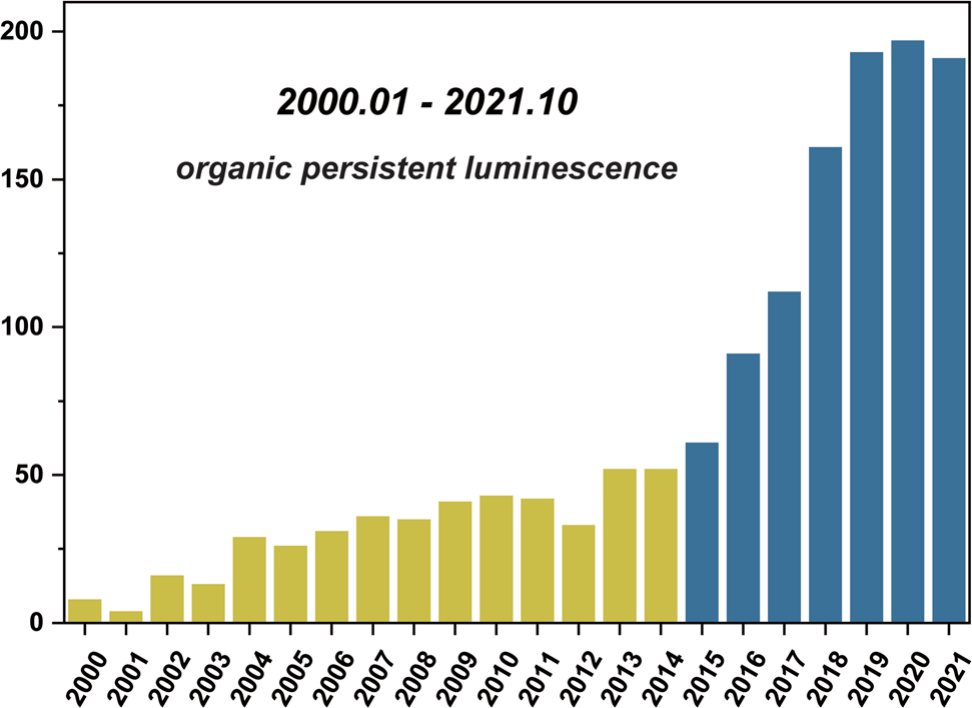
Statistical number of annual publications in the field of OPL emission (keywords: organic persistent luminescence, organic afterglow, organic ultralong emission, organic persistent emission or organic ultralong luminescence, not pollutant) from 2000 to 2021, data extracted from Web of Science.

Long persistent luminescence, which can be captured by the naked eyes even after removing the excitation light source, has received considerable attention in the applications of sensing, bioimaging, light emitting devices, and data encryption.^1–8^ Compared with the traditional transition metal-based or rare earth-based persistent luminescent materials, organic persistent luminescent (OPL) materials have become the research focus in recent years (Scheme 1) due to their advantages of flexible molecular design, easy synthesis, and low toxicity and production cost. The OPL properties are proved to be influenced greatly by the generation and deactivation process of the triplet excitons, including the intersystem crossing (ISC) process from singlet excited states to triplet excited states and the nonradiative decay and quenching processes. Slight variations in the surrounding microenvironment of OPL luminophores may influence these processes, leading to significant changes in the OPL emission intensity, colour, or lifetime. The stimulus-responsive characteristics indicate the great potential of OPL materials in environmental sensing, and the long lifetime characteristics amplify the response to external stimulation, increasing the sensitivity of OPL materials for sensing.^9,10^

Up to now, various methods have been developed to yield high-performance OPL materials, such as halogen substitution, crystallization, polymerization, host–guest doping and supramolecular assembly.^11–14^ Among them, halogen substitution has been proved to be one of the most effective design strategies.^15,16^ The introduction of different halogen elements will realize OPL emission with significantly tuneable intensity and lifetimes, making halide-containing OPL materials applicable to different environment sensing scenarios.^10,17^ In addition, halogen elements contribute to the formation of halogen bond networks, remarkably restricting molecular motion or vibration and boosting the OPL emission.^18^ Also, halogen bonding will yield an increase in the electron density of the halogens,^19^ promoting the heavy-atom effect and efficient ISC process.

To date, many halide-containing OPL materials have been reported, showing interesting applications in environmental sensing. Therefore, it is crucial for a systematic review of the material design and fundamental principles of halide-containing OPL materials for sensing applications. In this review, we summarize the current status of the development and sensing applications of halide-containing OPL materials, especially focusing on the superiority of these materials in environmental sensing, including the long emission lifetimes and high sensitivity. The photophysical processes and luminescence mechanisms of OPL materials are illustrated first to better understand the sensing mechanism and the advantages of halide-containing materials. Next, three typical halide-containing material systems exhibiting OPL emission are introduced, including small molecules, polymers, and doping systems. Subsequently, the novel utilization of these materials is summarized for the detection of temperature, oxygen, H_2_O, UV light and organic solvents. Finally, the personal view of the development of halide-containing OPL materials in the future is provided. This review focuses attention on the OPL emission and sensing mechanism and prefers the introduction of prompt representative halide-containing materials and interesting sensing applications, rather than the comprehensive discussion of this field.

## 2. Emission mechanisms of OPL materials

**Fig. 1:**
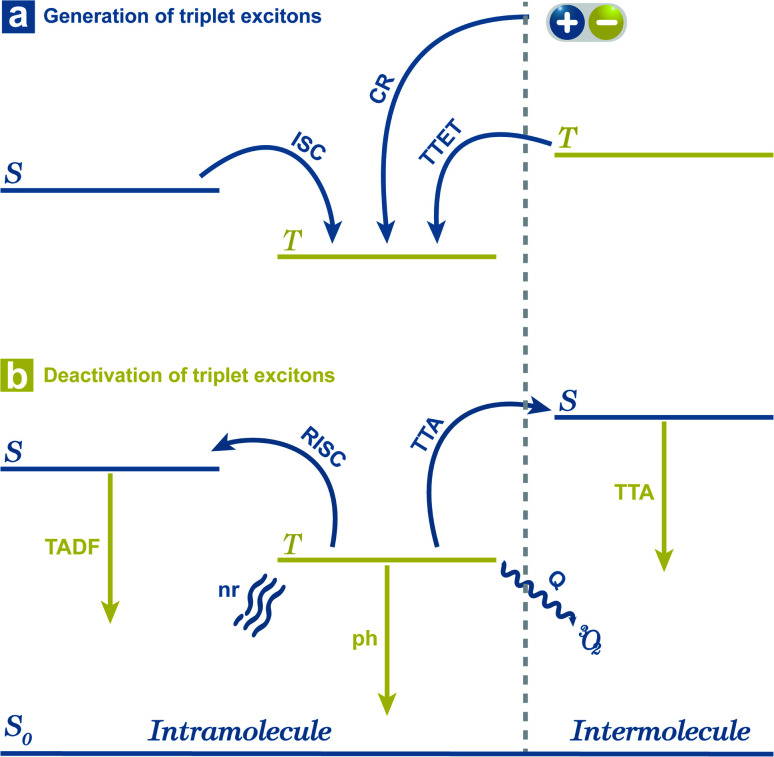
Simplified generation and deactivation process of triplet excitons. S stands for singlet excited states, and T stands for triplet excited states. 1: intersystem crossing (ISC); 2: charge recombination (CR) of electrons and holes; 3: triplet–triplet energy transfer (TTET); 4: reverse intersystem crossing (RISC); 5: thermally activated delayed fluorescence (TADF) emission; 6: phosphorescence (ph) emission; 7: triplet–triplet annihilation (TTA); 8: TTA emission; 9: nonradiative transition (nr); 10: oxygen quenching (Q) *via* energy transfer.

For the simplified room temperature phosphorescence (RTP) process in organic materials, the electrons in the singlet excited states undergo intersystem crossing to the triplet states, and then return to the ground states by the way of radiative transitions (phosphorescence) or nonradiative transitions. The effective production of triplet excitons is one crucial point deciding the efficiency of OPL materials. As shown in Fig. 1, the triplet excitons can also be generated *via* the charge recombination of electrons and holes (or the charge-separated state and photoinduced ionized state).^20,21^ In addition, triplet–triplet energy transfer (TTET) often occurs to the triplet states of acceptors from the donor molecules in multicomponent systems.^22^ Besides, a singlet exciton can also be converted to two triplet excitons by reacting with the neighbouring ground-state molecule, known as the singlet fission process.^23^ For single-component organic materials, the production of triplet excitons (*ϕ*_T_) is determined by the intersystem crossing rate (*k*_ISC_) according to eqn (1), and *k*_ISC_ between the singlet and triplet excited states (*S*_N_ and *T*_M_) can be given by eqn (2):^24,25^

1 *ϕ*_T_ = *k*_ISC_*τ*_S_

2
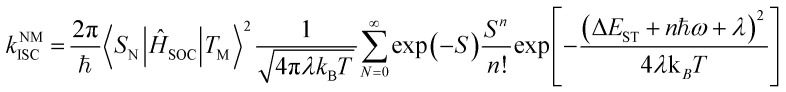
where 〈*S*_N_|*Ĥ*_SOC_|*T*_M_〉 and Δ*E*_ST_ are the spin–orbit coupling (SOC) elements and the energy difference between *S*_N_ and *T*_M_, ℏ is the reduced Planck's constant, *λ* is the Marcus reorganization energy, *k*_B_ is the Boltzmann constant, *T* is the temperature and *S* is the Huang–Rhys factor. Generally, SOC elements and the energy gap can be regulated by a rational molecular design strategy. For example, the introduction of heavy atoms, such as bromine and iodine, will enhance the SOC elements and the ISC process between *S*_N_ and *T*_M_, and the singlet and triplet excited states with charge transfer (CT) characteristics exhibit a small energy difference (Δ*E*_ST_).^26^ Besides, the ISC process will be enhanced if the process involves a change of orbital type, which is known as El-Sayed rules.^27^ Empirical formula (3) for estimating *k*^NM^_ISC_ in organic molecules without heavy atoms proves that *k*_ISC_ between n, π* and π, π* is increased by about two orders of magnitude than that between π, π* and π, π*:^28^3
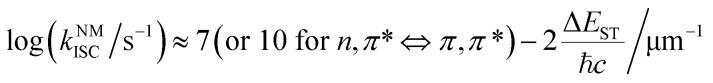
where ℏ is the Planck constant and *c* is the speed of light. Even so, efficient ISC processes can occur between two π, π* states with twisted molecular configuration for the non-zero SOC elements induced by the change in the orbital angular momentum.^29^

Corresponding with the generation, there are three radiative decay pathways for triplet excitons, including phosphorescence, thermally activated delayed fluorescence (TADF), and triplet–triplet annihilation (TTA) emission, which can all exhibit long emission lifetimes.^30–32^ In the case of small Δ*E*_ST_ or high temperature, endothermic reverse intersystem crossing (RISC) may occur from triplet to singlet excited states followed by TADF emission.^33^ Contrary to the singlet fission, TTA may occur between two neighbouring triplet excitons, leading to the generation of one singlet state and one ground state. Thus, TTA emission will lead to the loss of triplet excitons, and in most cases, the TTA mechanism is not recommended for the design of OPL materials.^29,34–37^ During the radiative process, reducing the nonradiative deactivation process of the triplet excitons is another point to achieve OPL emission, such as nonradiative transitions and external conversion. The nonradiative decay from the first triplet excited state (T_1_) to ground state (S_0_) is also regarded as the ISC process and the rate can be described by eqn (2). In this condition, the Marcus reorganization energy (*λ*) is the dominant factor in determining the nonradiative decay,^38^ and this process is presented as the random thermal vibrations of molecules. External conversion is a process in which the excited molecules interact with other molecules by collision or energy transfer and relax to the ground states. In particular, the ground state of molecular oxygen is the triplet state, leading to triplet excitons that are easily deactivated by sensitizing oxygen.^34,39^ Application of crystallization, self-assembly, host–guest interaction or polymer aggregation can suppress nonradiative decay and external conversion simultaneously to stabilize triplet excited states by weakening molecular vibration and reducing energy transfer to triplet oxygen.

## 3. Advantages of halide-containing OPL materials

For most pure organic compounds, the ISC process from singlet excited states to triplet excited states and the radiative transitions from the triplet excited states to singlet ground states are forbidden. Halogen substitution has been proved to be one of the most effective strategies to improve triplet exciton yields and OPL efficiency, and it is widely applied in the design and fabrication of OPL materials. Internal and external heavy-atom effects promote the ISC (or RISC) process and radiative transitions from triplet excited states to ground states, leading to distinct OPL intensity and lifetimes for halogen substituted compounds with different SOC constants.^7,8,29,40–44^ It brings great convenience to the OPL molecular design for different environmental sensing application. Generally, iodine-containing OPL materials exhibit high phosphorescence quantum yields with short lifetimes of about several milliseconds, while long emission lifetimes of about hundreds of milliseconds will be realized in chlorine substituted OPL derivatives. In addition, the formation of halogen bonds will promote OPL emission due to the significant suppression of molecular motion or vibration by halogen bonding.^15,45^ It has also been reported that halogen bonding may induce the partial electron delocalization of the electron donors to the halogen atoms, leading to the enhanced heavy-atom effect and efficient ISC process.^46–48^ Finally, the halogen substituents are better for realizing the rigid π–π stacking environment, specifically, face-to-face and edge-to-face stacking, which is common for aromatic derivatives.^49–51^

## 4. Design and classification of halide-containing OPL material systems

Several halide-containing aromatic compounds have been reported to exhibit phosphorescence emission by Lewis and Kasha in 1944.^52,53^ Meanwhile, the emission lifetimes of these halogen substituted compounds were measured to be on the order of seconds at 77 K by Mcclure, and the heavy atom effect on emission lifetimes has been studied.^54^ Kasha reported the external heavy-atom effect first by mixing α-chloronaphthalene and ethyl iodide.^55^ Since then, halogen substituents or halogen ions have been widely reported to enhance the production and radiative decay of the triplet excitons in organic phosphorescence materials and other OPL materials. In this section, we will introduce several typical halide-containing OPL material systems in recent years.

### 4.1 Small molecules

At present, many small molecules exhibiting OPL emission are developed,^11,13,56,57^ such as difluoroboron (BF_2_)–chelates,^58^ triazine,^59^ boronic esters,^60–62^ imide derivatives,^63^ and sulfones.^64,65^ Several representative small molecules are presented in this section, including the carbonyl compounds, carbazole compounds, organic halide salts, and sulfonyl derivatives.

#### 4.1.1 Carbonyl compounds

**Scheme 2:**
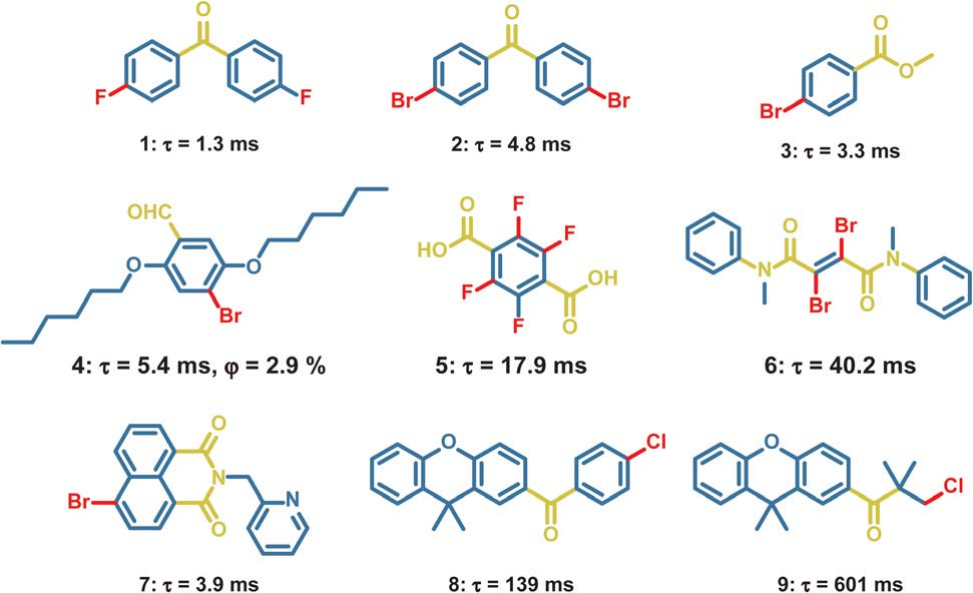
Chemical structure of halide-containing aromatic carbonyl molecules and their phosphorescence lifetimes and quantum yields.

Carbonyl groups have been proved to be efficient groups to promote phosphorescence. The lone-pair electrons in carbonyl groups contribute to the n, π* properties of the excited states, which will promote the ISC process between the n, π* and π, π* states and enhance the triplet exciton formation. OPL emission has been observed in a lot of carbonyl-containing materials, such as aldehydes, ketones, and carboxylate esters (Scheme 2). In 2010, Tang *et al.* reported the crystallization-induced phosphorescence of benzophenone derivatives and compounds 1–3 exhibit emission lifetimes of over 1 ms.^66^ Hydrogen bonding and halogen bonding in the crystals of 1–3 construct a rigid environment and restrict the molecular motions, leading to phosphorescence emission at room temperature. Also, Kim *et al.* realized phosphorescence emission in the crystal of compound 4.^45^ The halogen bonding in 4 reduces the distance between carbonyl oxygen atoms and bromine atoms, and promotes the radiative transition of triplet excitons. In addition, the carboxylic acid 5, amide 6 and naphthalimide 7 have been found to exhibit phosphorescence emission, but the OPL lifetimes of these materials are not larger than 100 ms.^67–69^ The OPL emission of the carbonyl-containing materials can last more than 0.1 s after the rational molecular design. Li *et al.* optimized the structures of 9,9-dimethylxanthene derivatives 8 and 9 by changing the rigid aromatic ring into a flexible alkyl chain, leading to more compact packing for 9 with an increased emission lifetime of 601 ms than 139 ms for 8.^70^ Yuasa *et al.* reported a series of benzoic acid derivatives with persistent TTA and phosphorescence emission. They demonstrated that singlet and triplet radical ion pairs were formed in the solid state with a marginal energy difference and hyperfine coupling promoted the singlet-to-triplet conversion, which both facilitated the formation of triplet excitons. In this case, the heavy atom effect did not act on the T_1_-generating ISC process and only 3-fluorobenzoic acid exhibits dual persistent luminescence.^37^

#### 4.1.2 Carbazole compounds

**Scheme 3:**
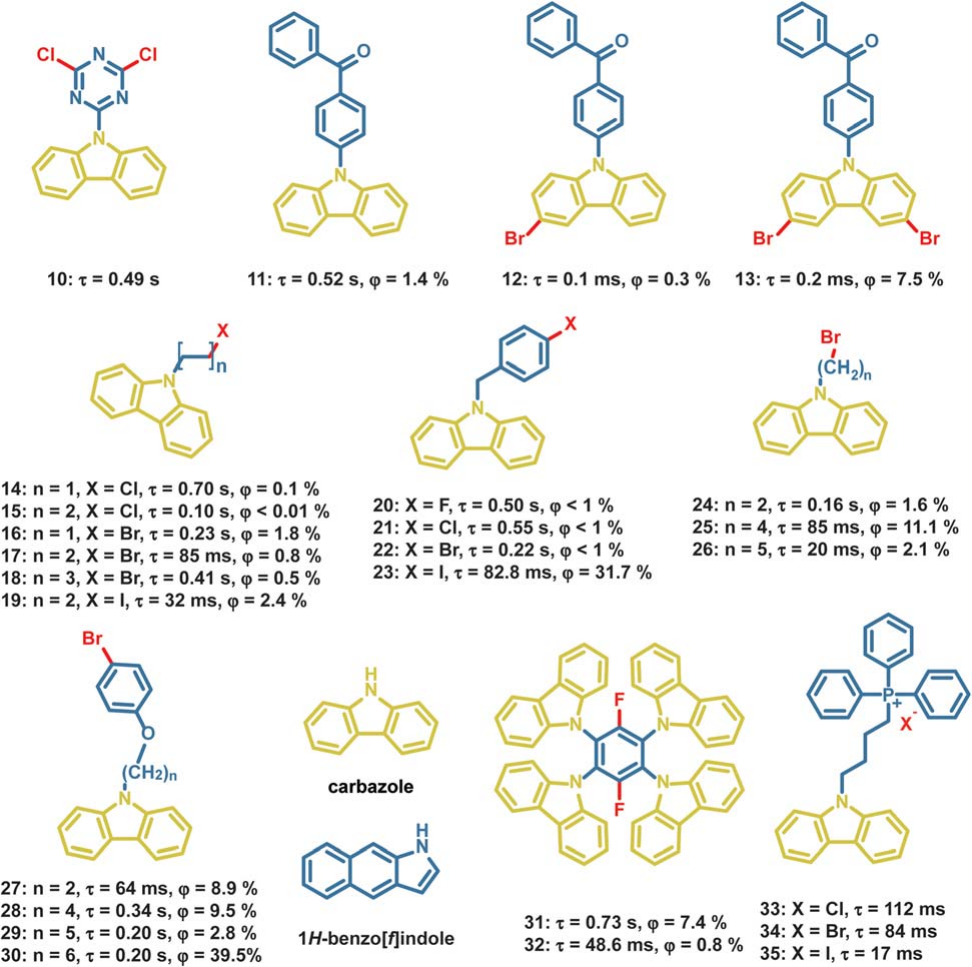
Chemical structure of halide-containing carbazole derivatives, and their phosphorescence lifetimes and quantum yields.

Besides, a large number of carbazole derivatives have been found to exhibit OPL emission (Scheme 3). In 2015, Huang *et al.* reported a series of carbazole derivatives with OPL emission, and the chlorine substituted compound 10 exhibits emission at 591 nm with a lifetime of 0.49 s.^71^ They put forward that the stable H-aggregation in the crystal facilitates the stabilization of the triplet excitons and contributes to the OPL emission. Tang *et al.* introduced bromine atoms in carbazole groups to get compounds 11–13 and found that the loose crystal packing and vigorous vibrational dissipation in compounds 12 and 13 led to no persistent emission at room temperature. The influence of different halogen atoms on the OPL emission of carbazole derivatives has been studied. The photophysical properties of compounds 14–23 indicate whether the halogen atom is on the aromatic ring or the alkyl chain, and the emission lifetime decreases and the OPL efficiency increases with the increasing halogen atomic number in most cases.^72,73^ In addition, the distance between halogen atoms and carbazole groups will influence OPL properties greatly. The flexible alkyl chains in compounds 24–30 prevent the intramolecular heavy atom effect of bromine, and induce a moderate heavy atom effect on the neighbouring luminophores, contributing to efficient OPL emission of 30.^74^ Recently, Liu *et al.* discovered that the isomeric impurity (1*H*-benz[*f*]indole) in carbazole significantly influenced OPL properties of carbazole derivatives,^75^ and Tang *et al.* also found that the lab-synthesized carbazole derivative 32 showed a shorter lifetime and lower OPL quantum yield than compound 31, which is synthesized with commercially purchased carbazole.^76^

#### 4.1.3 Organic halide salts

**Scheme 4:**
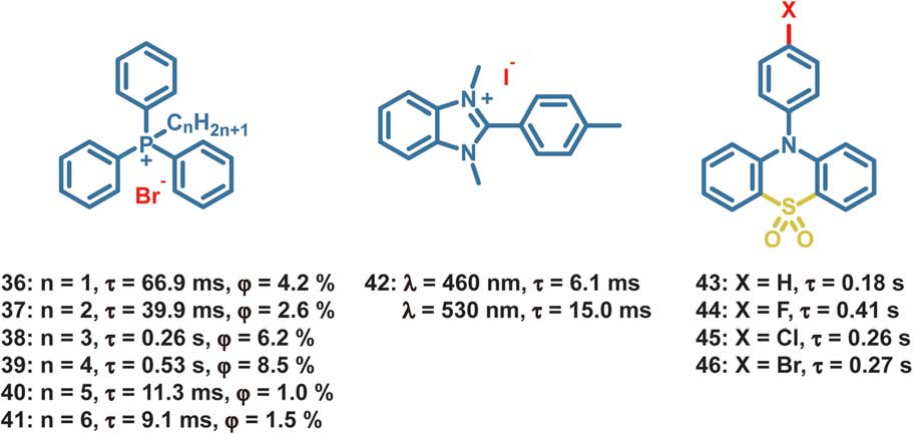
Chemical structure of halide ion complexes 36–42 and sulfonyl derivatives 43–46, and their OPL lifetimes and phosphorescence quantum yields.

In addition to the above common functional groups, halogen ions can be introduced in OPL materials directly to boost phosphorescent emission (Scheme 4), and the decrease of the OPL lifetime is observed with increasing halogen ion atomic number.^42^ The lifetime of phosphonium salt 33 is about six-fold longer than that of 35 due to the stronger external heavy-atom effect of iodide ions. Zhao and Huang also reported the effect of the alkyl chain length on the OPL properties of phosphonium salts 36–41 and their application in large-area security printing.^77^ Different alkyl chains will influence the tetragonal lattices in crystals, and the tighter molecular stacking is found in 38 and 39, leading to lower nonradiative transition rates, longer emission lifetimes and higher OPL efficiencies than 36 and 37. Gabriele *et al.* reported OPL properties of benzimidazolium salts 42 with triflate, nitrate or iodide anions, and they summarized the effects of ion-pairing on the photoluminescence properties.^78^ Weaker interaction is observed between the benzimidazolium cation and the triflate anion, leading to highly efficient fluorescence emission, while 42 shows intense molecular phosphorescence due to the heavy atom effect and efficient ISC process.

#### 4.1.4 Sulfonyl derivatives

The sulfonyl group is also considered to be an effective group to induce OPL emission.^79–83^ Li and Pu *et al.* reported the OPL properties and the imaging application of several 10-phenyl-10*H*-phenothiazine-5,5-dioxide-based derivatives 43–46.^51^ The lone pair electrons of O and N atoms in 43–46 promote the ISC process according to the El-Sayed rules. Also, the introduction of electron-withdrawing halogen atoms (Br, Cl, and F) into the aromatic rings decreases the electron density of π-rings and relieves the π–π repulsion, leading to strong π–π packing and ultralong OPL lifetimes.

### 4.2 Polymers

**Scheme 5:**
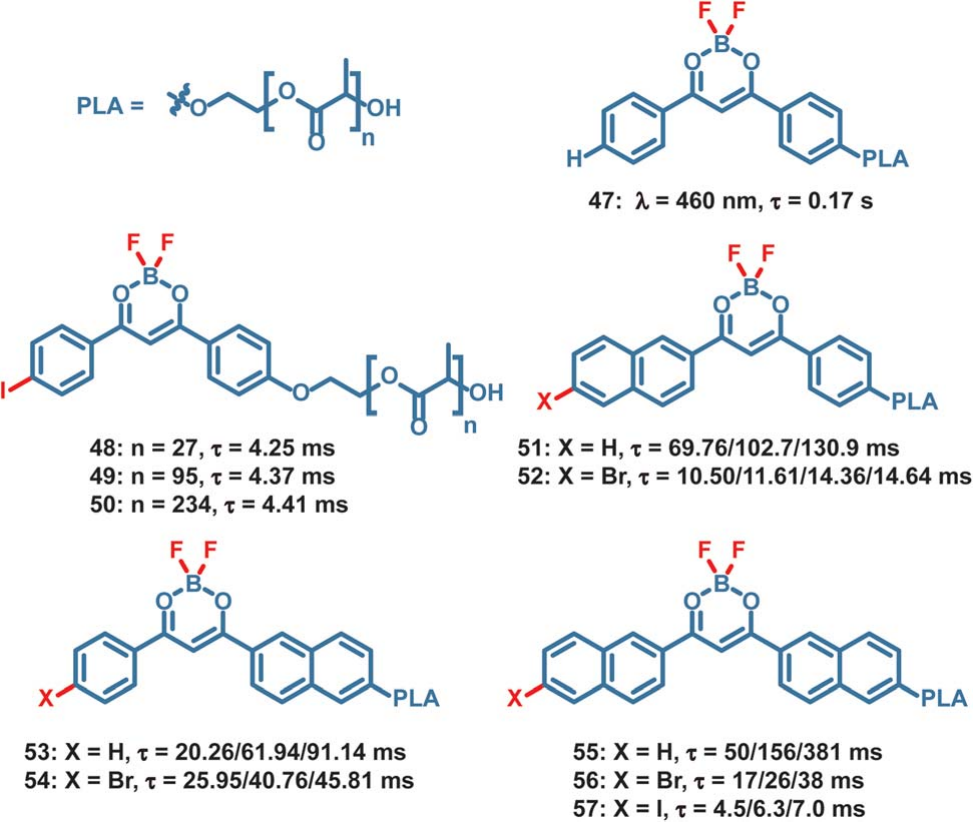
Chemical structure of BF_2_ polymers and their phosphorescence lifetimes. The polymer films with different molecular weights exhibit different lifetimes.

Compared to small molecule OPL materials, it is much easier to regulate the optical properties of OPL polymers by changing the molecular weight, monomer ratio and polymer types. Also, the polymer matrix itself provides a rigid environment to restrict the chromophore and exclude oxygen, leading to efficient photoluminescence. Unlike most small molecules only showing OPL emission in the crystalline state, OPL polymers can be prepared as films, powder, and nanoparticles, exhibiting wider application ranges. Fraser *et al.* reported a series of difluoroboron (BF_2_) polymers (Scheme 5, 47–57) coupled with poly(lactic acid) (PLA), and OPL emission can be observed from these polymer films.^10,17,84–86^ They found that the photophysical properties can be easily tuned using the molecular weight of the polymers, including the wavelength and lifetimes of the fluorescence and phosphorescence emission. With the increase of the molecular weight, a significant increase of the OPL lifetime can be found for the polymers 51–57.

**Scheme 6:**
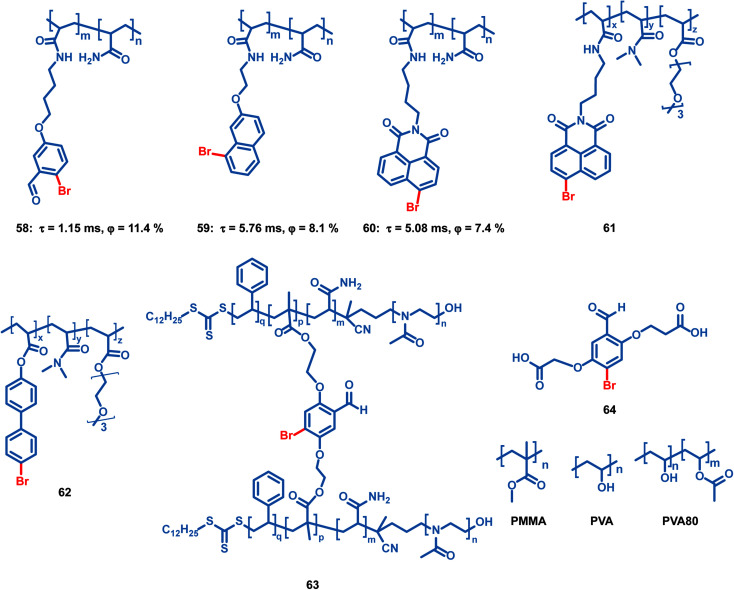
Chemical structure of bromine-containing copolymers and their phosphorescence lifetimes and quantum yields.

Tian *et al.* reported several OPL copolymers 58–62 (Scheme 6) containing different brominated aromatic compounds with polyacrylamide or poly(ethyl acrylate) as the polymer matrix.^87,88^ The heavy atom effect of bromine promotes the phosphorescence efficiency, and various phosphorescence colours from green to orange-red are realized by changing the bromine-containing phosphor from benzaldehyde to 1,8-naphthalic anhydride. Also, the polymers 55–57 retain the phosphorescence emission in the solvent and they can be used for water detection.^88^ Kim *et al.* reported core–shell polymer nanoparticles 63 with bromine-containing phosphor 64 as the phosphorescence chromophore and the water-soluble poly(2-methyl-2-oxazoline) as the outer shell.^89^ The short and simple crosslinking agent effectively restricts the molecular movement of the phosphor and polymer matrix, thereby effectively inhibiting the nonradiative relaxation pathway and helping to achieve a high phosphorescence quantum efficiency of about 40%. The nanoparticles show high sensitivity to oxygen, and they can be used for the monitoring of dissolved oxygen in aqueous media.

### 4.3 Doping systems

Like the OPL polymers, the host matrix in doping systems provides a rigid environment for the luminous guest, avoiding the significant nonradiative transition and oxygen quenching.^12^ Kim *et al.* reported simple doping of phosphor 4 and 64 into polymers polymethyl methacrylate (PMMA), polyvinyl alcohol (PVA) or 80% hydrolysed polyvinyl alcohol (PVA80). These doped polymer films exhibit phosphorescence emission under ambient conditions, but the phosphorescence quantum efficiency is much lower than that of copolymer 63 due to the significant suppression of the cross-linker.^89–91^ Also, the charge transfer process in the donor–acceptor doping system can promote the production of long-lived excitons, leading to persistent luminescence. Adachi and Kabe *et al.* reported a series of doping materials based on the mixture of electron-donating and electron-accepting molecules, exhibiting OPL emission lasting for more than one hour.^21,32,92^

**Scheme 7:**
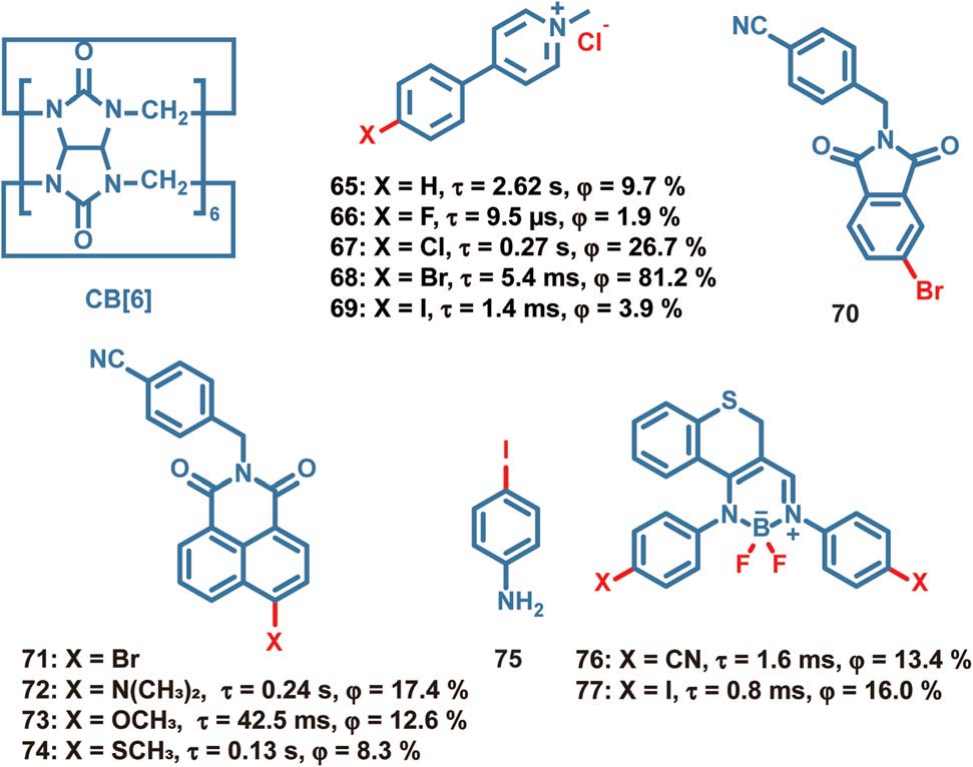
Chemical structure of guest molecules 65–69, 72–74, 76 and 77, and host molecules CB[6], 70 and 75. Phosphorescence lifetimes and quantum yields of 65–69 doped in CB[6], 72–74 doped in 70, and 76 and 77 doped in 75 are listed.

Liu *et al.* reported a series of OPL supramolecules with the cucurbit[6]uril (CB[6]) as the host and the pyridine salts 65–69 (Scheme 7) as the guest.^48,93^ Single-component 4-(4-bromophenyl)-*N*-methylpyridinium salts with different counterions (Cl^−^, Br^−^, I^−^ and PF_6_^−^) exhibit similar lifetimes of about 6 μs, but the bonding C–Br⋯I^−^ induces the electron delocalization of I^−^, leading to enhanced spin–orbit coupling and intense phosphorescence emission.^47,48^ These compounds are then embedded in CB[6], and sharp promotion of phosphorescence emission was observed except I^−^ counterions. The pyridine salts are deeply encapsulated in the CB[6] cavity, and the strong host–guest interaction suppresses the nonradiative decay seriously. Also, they introduced different halogen substituents in the chromophore pyridine salt to tune the lifetimes and achieve outstanding OPL emission for the supramolecule composed of pyridine salt 67 and CB[6] with a quantum efficiency of 26.7%.^93^

Zhang and Huang *et al.* reported OPL emission from a binary doping system with 70 as the host molecule even at a guest-to-host ratio of 10 parts per billion.^94^ The efficient TTET process from the delocalized host aggregates to the guest naphthalimide 72–74 contributes to the intense OPL emission. It is notable that the external heavy-atom effect of the host molecule is not the dominant cause of OPL emission, because no phosphorescence is observed with bromine substituted naphthalimide 71 as the host molecule. Fu *et al.* doped the chromophore BF_2_ derivatives 76 and 77 into the host crystalline matrix NH_2_–Ph–I 75, and the halogen bonding CN⋯I–Ph–NH_2_ and I⋯NH_2_–Ph–I contributes to the OPL emission of the doping systems.^46^ Also, the enhanced heavy atom effect of iodine was found in 77/75 (the former material is the guest material, and the latter one is the host material) because the I⋯NH_2_–Ph–I interaction induces the partial electron delocalization of the nitrogen to the iodine, resulting in an efficient ISC process in chromophore 77.

To sum up, efficient OPL emission with long lifetimes can be achieved in different halide-containing material systems. Generally, small molecules show good crystallinity and certain crystal structures are helpful for better understanding OPL mechanisms, which provide a theoretical basis for designing other OPL material systems. Polymers exhibit superiorities in flexible design and easy functionalization, and especially they can be prepared as water-soluble nanoparticles, which are necessary for the sensing application in aqueous or biological environments. Similarly, the flexible material design of doping systems makes it easy to realize colourful OPL emission by changing the guest molecules.^95,96^ Remarkably, ultralong luminescence with emission lifetimes greater than 1 hour can be achieved in pure organic doping systems.^32,92,97–99^

## 5. Sensing mechanism of OPL materials

The lifetime and quantum yield of phosphorescence and TADF can be expressed as eqn (4)–(7):^13,25,26,38,100,101^4
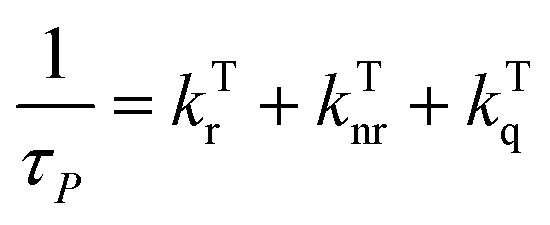

5 *φ*_P_ = *φ*_ISC_*k*^T^_r_*τ*_P_ = *φ*_ISC_[1 − (*k*^T^_nr_ + *k*^T^_q_)*τ*_P_]

6
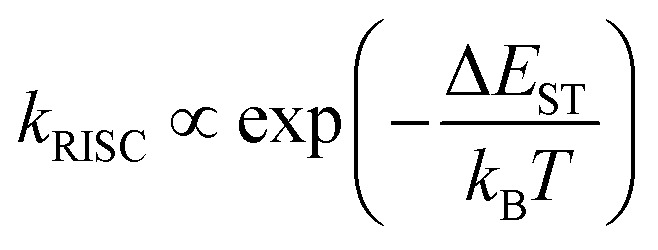
7
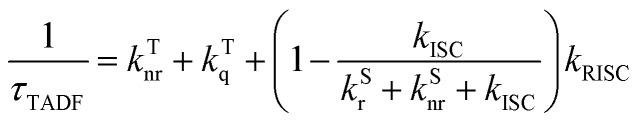
where superscripts S and T refer to the singlet and triplet excited states, respectively; *τ*_TADF_ and *τ*_P_ are the lifetimes of TADF and phosphorescence; *k*_ISC_ and *k*_RISC_ are the rate constants of ISC and the reverse intersystem crossing (RISC) process; *k*^S^_r_, *k*^S^_nr_, *k*^T^_r_ and *k*^T^_nr_ are the rate constants of radiative transition (r), nonradiative transition (nr), respectively, and *k*^T^_q_ is the quenching rate coefficient of triplet states by the environmental quenchers. Nonradiative transition and the RISC process are endothermic processes,^26,102^ which are strongly affected by temperature, leading to significant difference in phosphorescence and TADF emission at distinct temperatures. In addition, the kinetics of OPL emission quenching can be expressed as the Stern–Volmer relationship:^9,10,103^8
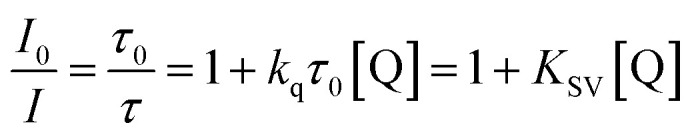
where *I*_0_ and *τ*_0_ are the OPL emission intensity and lifetime without the quencher, *I* and *τ* are the intensity and lifetime with the quencher, [Q] is the concentration of the quencher, and *k*_q_ and *K*_SV_ are the quenching rate coefficient and the Stern–Volmer quenching constant. The reaction probability of the triplet excitons of OPL materials with quenchers increases significantly for the longer lifetimes compared to the traditional phosphorescent transition metal complexes, and larger *τ*_0_ and *K*_SV_ indicate that the great change in the OPL intensity and lifetimes can be induced by low concentration quenchers. Thus, compared with classical fluorescent and phosphorescent sensors with emission lifetimes on the nanosecond or microsecond scale, the intensity and lifetimes of OPL emission are more sensitive to environmental changes, such as temperature, oxygen concentration and water.

OPL emission heavily relies on the molecular stacking or rigid matrices, and the breaking of the rigid environment of the phosphors may induce a decrease in the OPL emission intensity and lifetimes. For instance, the polymer matrices may be dissolved in water or organic solvents, leading to the OPL quenching of phosphors, which can be used for the detection of specific species. Similarly, the crystalline packing may be changed upon exposure to solvent molecules, such as HCl and CHCl_3_, contributing to the change of luminescence properties. Compared with OPL emission, temperature or oxygen has little effects on fluorescence emission, leading to the realization of wavelength-ratiometric probes for sensing temperature or oxygen based on the changes in the ratio of OPL and fluorescence emission intensity. Therefore, high-efficiency OPL emission can be achieved in well-designed halide-containing materials with disparate lifetimes ranging from several milliseconds to hundreds of milliseconds or seconds to satisfy different environmental sensing applications.

## 6. Various environmental sensing applications

In consideration of the unique emission properties of OPL materials, they have been widely applied in emerging technologies, such as anti-counterfeiting, data encryption, display, sensing, bioimaging and many others. In particular, the long emission lifetimes of OPL materials make them more sensitive to external stimulation than short-lived fluorescent materials, proving their superiority in environmental sensing applications. Also, the ultralong emission lifetimes of OPL materials can be distinguished using a cost-effective camera, making it possible for quantitative detection in environmental sensing without professional and expensive equipment. In this section, we will describe the novel utilization of halide-containing OPL materials in sensing temperature, oxygen, H_2_O, UV light and organic solvents.

### 6.1 Temperature sensing

**Fig. 2:**
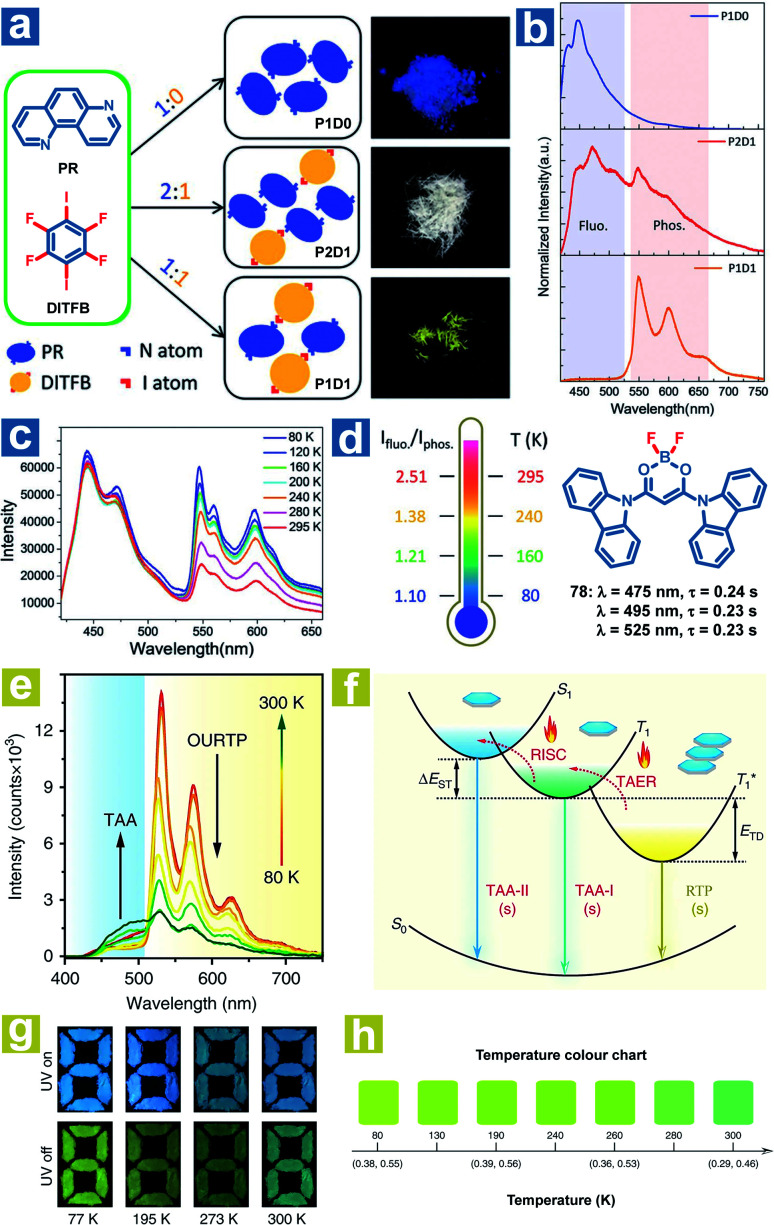
(a) Schematic illustration of the molecular packing and the photographs of P1D0, P2D1 and P1D1 cocrystals formed by cocrystallization of PR and DITFB at different ratios (1 : 0, 2 : 1, and 1 : 1). (b) Photoluminescence spectra of P1D0, P2D1 and P1D1 cocrystals. (c) Photoluminescence spectra of P2D1 cocrystals at different temperatures. (d) The ratio of fluorescence and phosphorescence intensity of P2D1 cocrystals at different temperatures. (e) Temperature-dependent phosphorescence spectra of 78 crystals from 80 to 300 K. (f) Schematic illustration of the temperature-dependent tri-mode OPL emission. (g) Photographs of the pattern filled with 78 powder before and after UV excitation at different temperatures. (h) OPL colour chart with the corresponding CIE coordinates of 78 crystals at different temperatures. (a–d) Reproduced from ref. 104 with permission from Copyright 2019, WILEY-VCH; (e–h) reproduced from ref. 31 with permission from Copyright 2020, Springer Nature.

According to eqn (4)–(7), the OPL emission intensity and lifetimes change upon temperature variation. Particularly, TADF and phosphorescence exhibit opposite trends in the emission intensity, leading to the change of OPL colours at distinct temperatures. Thus, the temperature can be detected using the OPL emission colours and lifetimes. Fu *et al.* demonstrated a cocrystal as a ratio thermometer using the relationship between the temperature and the intensity ratio of fluorescence and phosphorescence.^104^ As shown in Fig. 2, they found that the phosphorescence intensity of cocrystals increased with the stoichiometry of 1,4-diiodotetrafluorobenzene (DITFB) in 1,7-phenanthroline (PR). I⋯N halogen bonds in P1D1 cocrystals are twice more than those in P2D1 cocrystals, leading to faster rates of ISC and phosphorescence emission. Also, more I⋯N halogen bonds and stronger F⋯F interactions in P1D1 cocrystal contribute to a more rigid structure with a 3D network in P1D1 cocrystals than that in P2D1 cocrystals. The moderate fluorescence and phosphorescence emission made the P2D1 cocrystals possible to be a ratio thermometer (Fig. 2c and d). As the temperature increases from 80 to 295 K, there is no obvious change in fluorescence emission with a continuous decrease in phosphorescence emission intensity, and the ratio of dual emission intensity can be used for sensing temperature.

Huang and Chen *et al.* reported highly efficient tri-mode OPL emission in BF_2_ derivatives 78 (Fig. 2).^31^ As shown in Fig. 2e, one emission is the molecular phosphorescence emission at 495 nm with a lifetime of 234 ms, one is the TADF emission at 475 nm with a lifetime of 240 ms, and the last one is the phosphorescence emission from the stabilized triplet excited state at 525 nm with the lifetime of 232 ms. Efficient thermally activated afterglow (TAA) emission can be realized by the cascade thermally activated processes through releasing the long-lived 
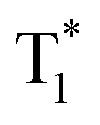
 excitons and transforming the spin-forbidden phosphorescence of 
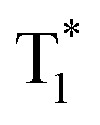
 and T_1_ emission into the spin-allowed emission of S_1_. The BF_2_ group serves as the acceptor, contributing to the CT characteristics and small Δ*E*_ST_. The endothermic characteristics of TAA emission contribute to the temperature-dependent OPL emission colours from 78, leading to the various afterglow colours of the pattern from blue-green at 300 K to green-yellow at 77 K (Fig. 2g). Also, the OPL colour chart (Fig. 2h) with the corresponding CIE coordinates at different temperatures indicates the potential application of 78 in the quantitative visual detection of temperature. Dong *et al.* reported a series of novel host–guest organic phosphors capable of dynamic OPL colour modulation from cyan (502 nm) to orange-red (608 nm).^105^ They cleverly take advantage of the quite different sensitivity of various doping materials to temperature to prepare three-component doped materials. The three-component system shows excellent phosphorescent thermochromic properties, that is, the material shows green phosphorescence at room temperature, and the doped material gradually exhibits orange phosphorescence after the temperature increases.

**Fig. 3:**
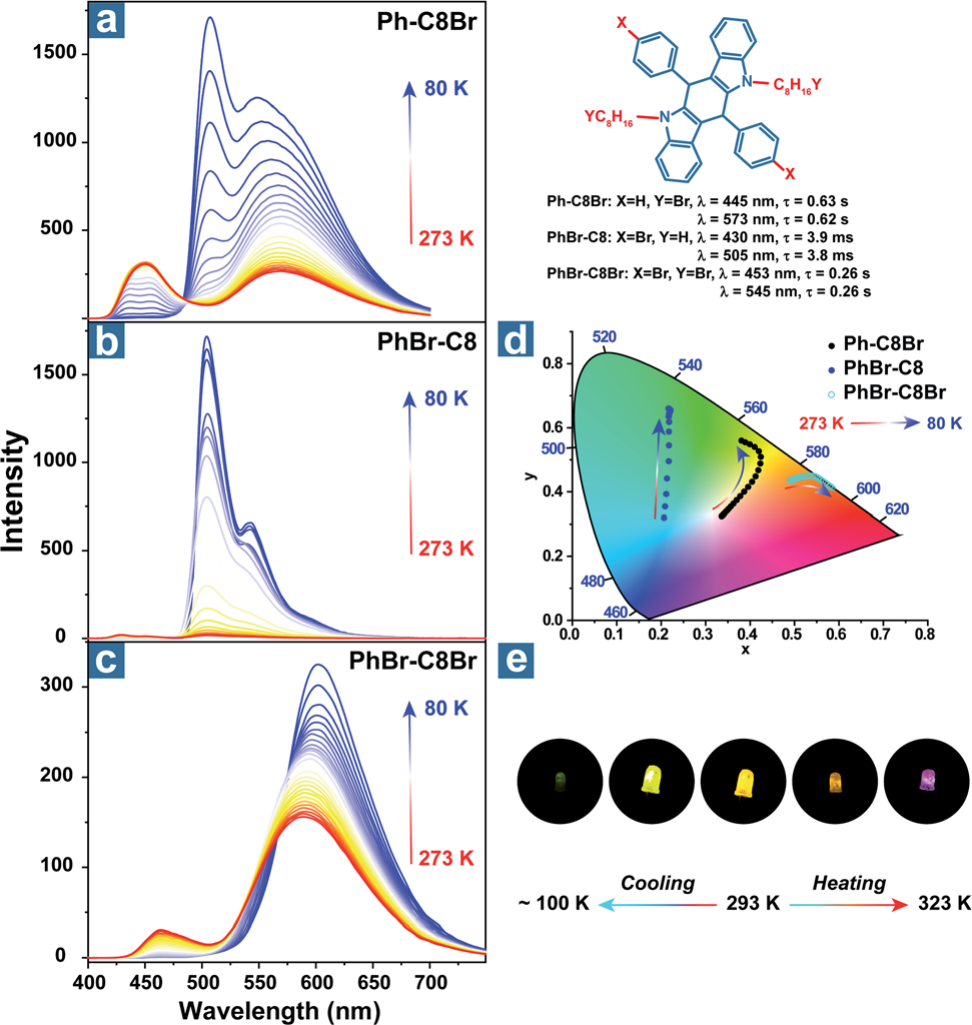
Temperature-dependent delayed emission spectra of (a) Ph-C8Br, (b) PhBr-C8 and (c) PhBr-C8Br. (d) The CIE 1931 coordinates of delayed emission at different temperatures. (e) After glow photographs of the UV-LED coated with Ph-C8Br at various temperatures. Reproduced from ref. 106 with permission from Copyright 2021, WILEY-VCH.

Zhao *et al.* reported the temperature sensing application based on the persistent TADF and phosphorescence emission.^106^ As shown in Fig. 3, bromine atoms were introduced into the aromatic rings and alkyl chains to yield Ph-C8Br, PhBr-C8 and PhBr-C8Br, which all exhibited dual persistent luminescence and significantly various OPL colours at different temperatures. Also, dual emission can be regulated by two halogen substitution strategies at different positions. Aromatic bromine atoms contribute to the CT transition between the distorted polycyclic and benzene rings, boosting the RISC process and TADF emission. Instead, halogen substitution at the terminal carbon atoms suppresses the alkyl chain vibration and exhibits external heavy-atom effects on the adjacent chromophores, leading to efficient persistent phosphorescence emission for Ph-C8Br and PhBr-C8Br. Ph-C8Br was then coated on the commercial UV LED, and it dominantly showed green phosphorescence afterglow at low temperature and persistent TADF emission after heating, indicating the feasibility of the OPL materials in temperature sensing.

**Fig. 4:**
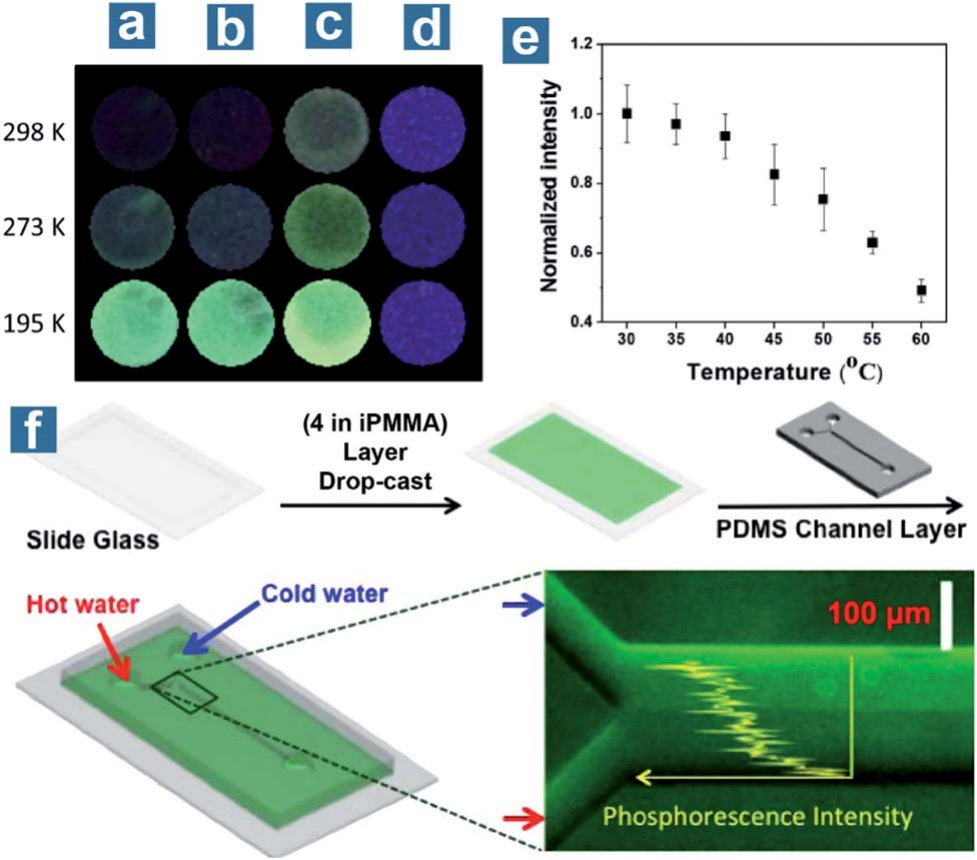
Photoluminescence emission of 4 embedded in (a) aPMMA, (b) sPMMA, and (c) iPMMA and (d) pure 4 at different temperatures. (e) Normalized phosphorescence emission intensities for 4 embedded in iPMMA at different temperatures. (f) Fabrication process, operation and the fluorescence microscope image of the temperature sensing devices based on the phosphorescent polymer (4 in iPMMA). *λ*_ex_ = 365 nm. Reproduced from ref. 107 with permission from Copyright 2013, American Chemical Society.

Also, Kim *et al.* reported the application of the doping system in sensing temperature with compound 4 embedded in the host polymer PMMA.^107^ They found that the doping polymers exhibited different quantum efficiency in atactic, isotactic and syndiotactic PMMA (aPMMA, iPMMA, sPMMA) with various polymer tacticity. β-relaxation, caused by the onset of rotation of the ester side group in PMMA, is regarded as the reason for the low phosphorescence quantum efficiency in aPMMA and sPMMA. The intensity of the β-relaxation decreases in iPMMA, with larger isotacticity than in aPMMA and sPMMA, leading to the locked carbonyl dipole motion and effective OPL emission (Fig. 4a–d). Phosphorescence intensity changes linearly with temperature at about the glass transition point of iPMMA, 55 °C, at which temperature long-range segmental motions in polymers occur (Fig. 4e). As shown in Fig. 4f, a microfluidic device is fabricated composed of a polydimethylsiloxane (PDMS) channel on a glass slide coated with 4 embedded in iPMMA. Hot (60 °C) and cold (4 °C) water is injected through two inlets into the microchannel, respectively, to generate laminar flow inside the channel with a thermal gradient induced at the boundary. The fluorescence microscope image proves a linear decrease of phosphorescence emission intensity along the thermal gradient from the cold to hot side.

### 6.2 Oxygen sensing

**Fig. 5:**
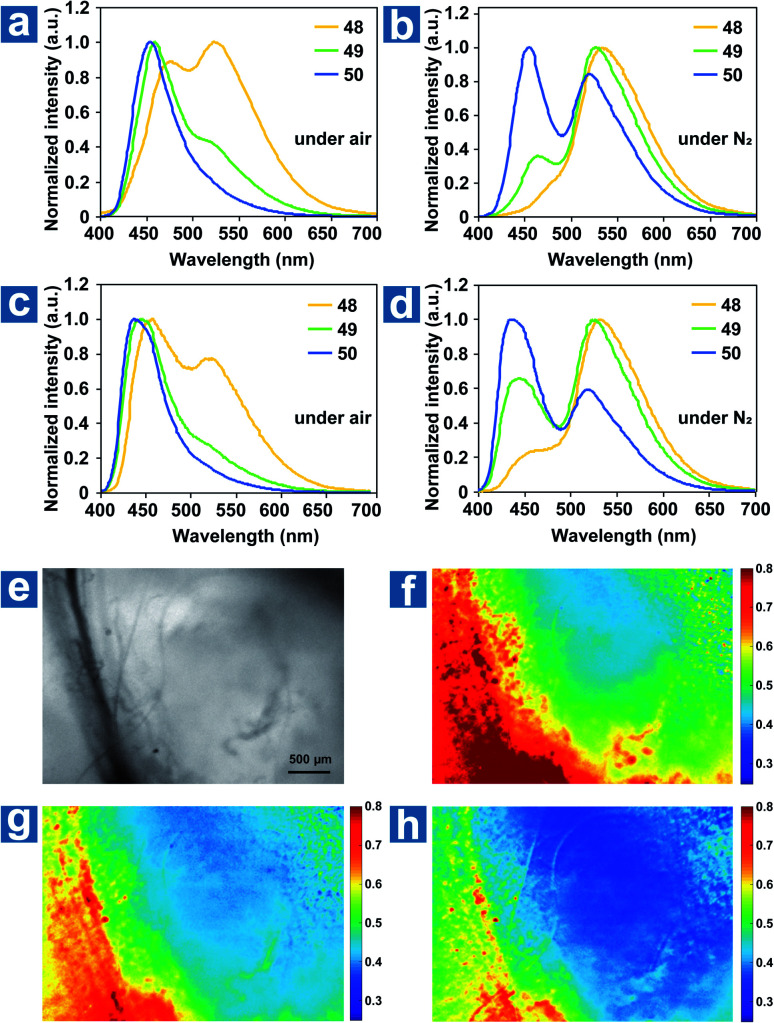
Photoluminescence spectra of polymers 48–50 as powders (a and b) and spin-cast films (c and d) under air and under N_2_. (e–h) Hypoxia imaging of the breast cancer 4 T1 mammary carcinoma tumour region in a mouse window chamber model with 49 nanoparticles. The bright-field (e) and nanoparticle fluorescence/phosphorescence ratio while breathing carbogen-95% O_2_ (b), room air-21% O_2_ (c) and nitrogen-0% O_2_ (d). The blood vessel is on the left side of the image (yellow-red regions in the ratio images), and the right region is the tumour (blue regions in the ratio images). Reproduced from ref. 17 with permission from Copyright 2009, Nature Publishing Group.

The ground state of molecular oxygen is the triplet state, making it a powerful quencher of triplet excitons for the spin-allowed energy transfer from OPL materials to oxygen. Thus, according to eqn (8), OPL materials show different emission intensity and lifetimes at distinct oxygen concentrations. Zhang and Fraser *et al.* reported a series of BF_2_ derivatives coupled with poly(lactic acid) (PLA), showing the unusual and long-lived OPL emission in the absence of O_2_.^17,84^ They controlled the heavy atom content in the polymers by introducing variable PLA chains to modulate the fluorescence and OPL emission (48–50).^17^ As shown in Fig. 5a–d, the phosphorescence intensity increases with the increase of the iodine atom content in the polymer. Also, the red-shifted fluorescence band at about 480 nm is found in the low-molecular-weight polymer 48, in comparison with 470 nm in 49 and 456 nm in 50. The energy gap between the singlet and triplet states decreases with the molecular weight, contributing to the enhanced ISC and the boost of OPL emission according to eqn (2). A linear dependency is found between the ratio of the fluorescence/OPL emission and the oxygen levels (up to 1% O_2_) in the film 48, making it a “turn on” sensor in low-oxygen environments. Due to the comparable and balanced fluorescence and phosphorescence intensities of 49, it is fabricated as nanoparticles (NPs) by nanoprecipitation to be ratiometric tumour imaging agents under 95, 21 and 0% O_2_ (Fig. 5e and f). Excellent contrast between the microvasculature (red) and the hypoxia tumour tissue (blue) proves the potential of the compound in oxygen sensing.

**Fig. 6:**
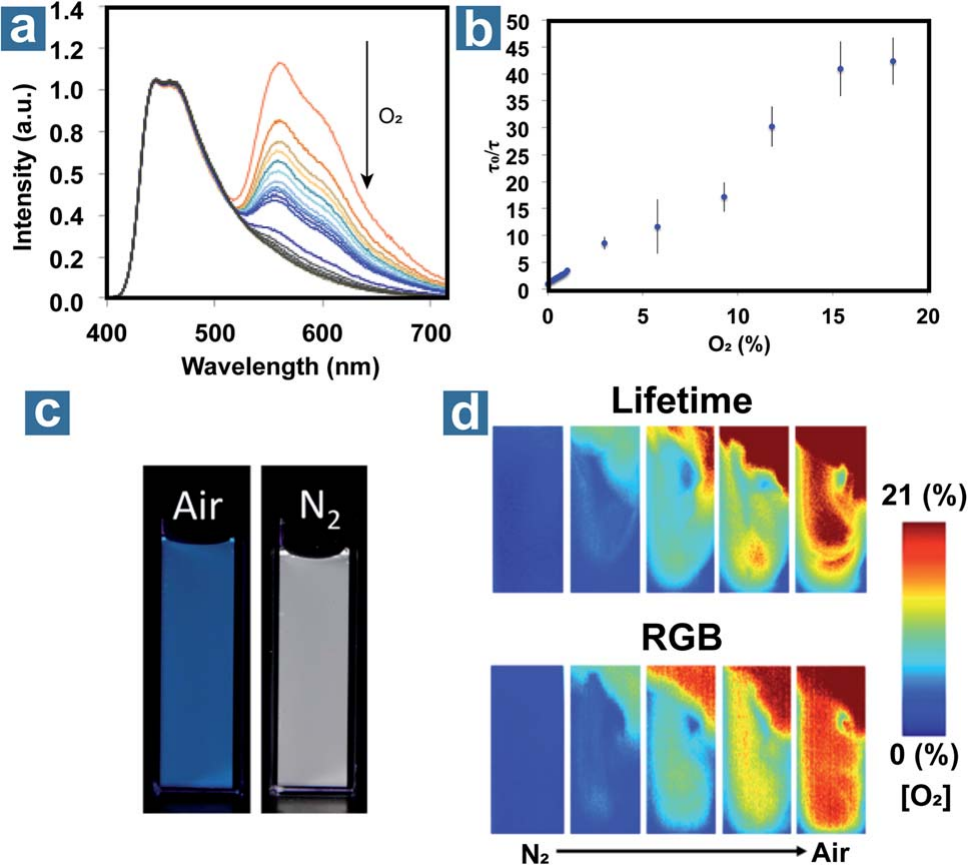
(a) Photoluminescence spectra of the nanoparticle 52 at different oxygen concentrations. (b) Lifetime Stern–Volmer plot of the nanoparticle 52. (c) Images of the nanoparticle 52 in N_2_ and air. (d) Dual-mode imaging of oxygen sensing based on the lifetime and emission colour. Reproduced from ref. 10 with permission from Copyright 2016, American Chemical Society.

Fraser *et al.* optimized the BF_2_ polymers to obtain the materials 51–52 with the dual emission at about 460 nm and 560 nm.^10^ Based on the changed emission colours and lifetimes at different oxygen concentrations, the oxygen levels can be quantified using lifetimes and red/green/blue (RGB) ratiometry using a portable and cost-effective camera. Fig. 6 shows the simultaneous dual-mode oxygen sensing by compound 52. Due to intense RTP emission from nanoparticle 52, the RGB channels of the camera can be used to independently monitor changes in fluorescence (F) and phosphorescence (P) emission for referenced (F/P) oxygen sensing, employing the blue channel for the reference (F) and the red channel as the sensor (P). As shown in Fig. 6d, dual-mode imaging presents the bubbling process of air (21% O_2_) into the solution of 52, which is purged with N_2_ in advance. Oxygen levels measured by the two methods are in good agreement with each other, and imaging based on OPL lifetimes shows higher spatial resolution than that based on RGB methods. This may result from the weak OPL emission at high oxygen concentration, which will suffer from the interference of fluorescence emission.

**Fig. 7:**
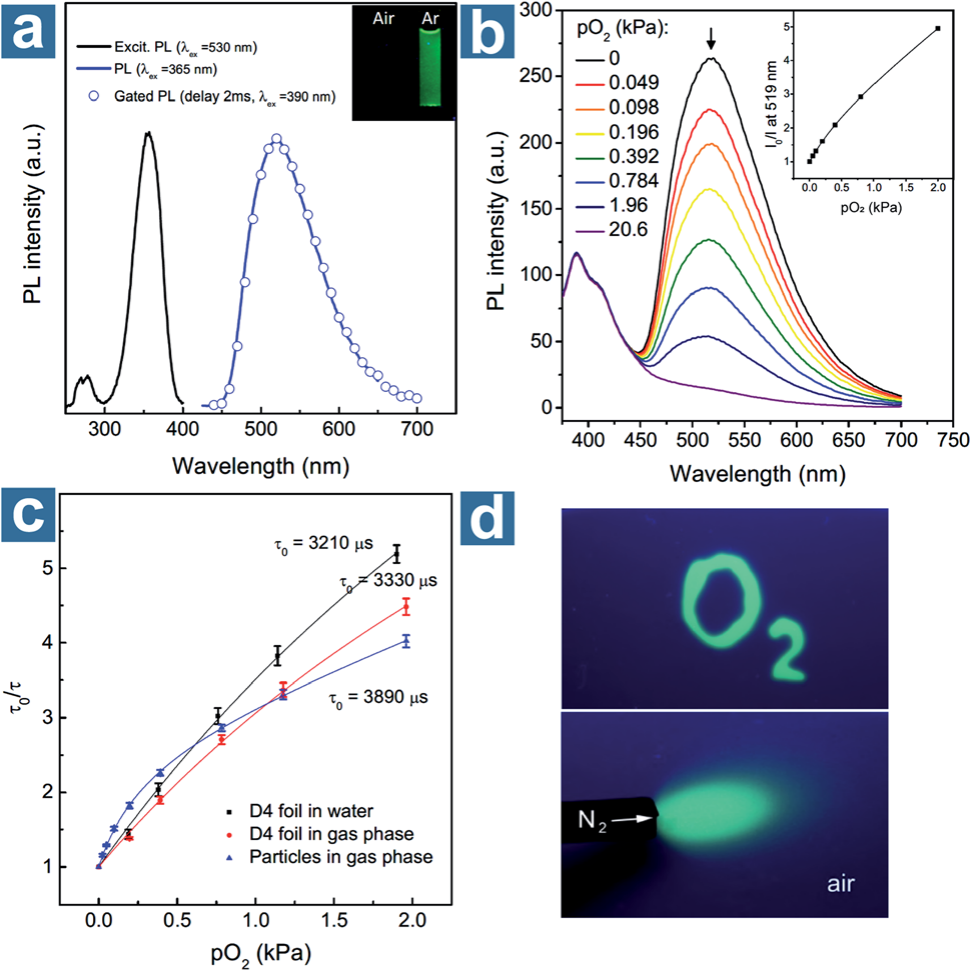
(a) Photoluminescence emission, excitation, and gated photoluminescence spectra of nanoparticle 63 in argon saturated water (*c* = 10 mg mL^−1^). (b) Photoluminescence spectra of nanoparticle 63 in polyurethane hydrogel D4 at different oxygen pressures and the intensity Stern–Volmer plots (*λ*_ex_ = 365 nm, *λ*_em_ = 519 nm). (c) Lifetime Stern–Volmer plots of the dry nanoparticle 63 in the gas phase, and the sensor based on 320 nm nanoparticle 63 dispersed in polyurethane hydrogel D4 in the gas phase and in water. (d) Optical images of the sensor under the excitation of a 365 nm UV lamp. The upper bright green region was soaked with an anoxic aqueous solution (containing 5% wt of glucose and 0.05% wt of glucose oxidase). The below green area was deoxygenated with a flow of nitrogen. Reproduced from ref. 91 with permission from Copyright 2017, Wiley-VCH.

Kim *et al.* devised a sensitive oxygen detection platform based on crosslinked core–shell polymeric nanoparticle 63 with bromobenzaldehyde phosphors.^91^ As shown in Fig. 7a, no essential emission is observed in aqueous solution of nanoparticle 63, but it shows bright green emission after the elimination of oxygen by argon purging. The relationship between the lifetime *τ*_0_/τ and oxygen pressure pO_2_ is measured to quantify the sensitivity of NPs to oxygen in gaseous phase and in water. The Stern–Volmer plots show a near-linear behaviour and the limit of detection of dissolved oxygen is estimated to be 60 nM in aqueous environments, indicating the sensitive detection of dissolved oxygen in a variety of environments.

### 6.3 H_2_O sensing

**Fig. 8:**
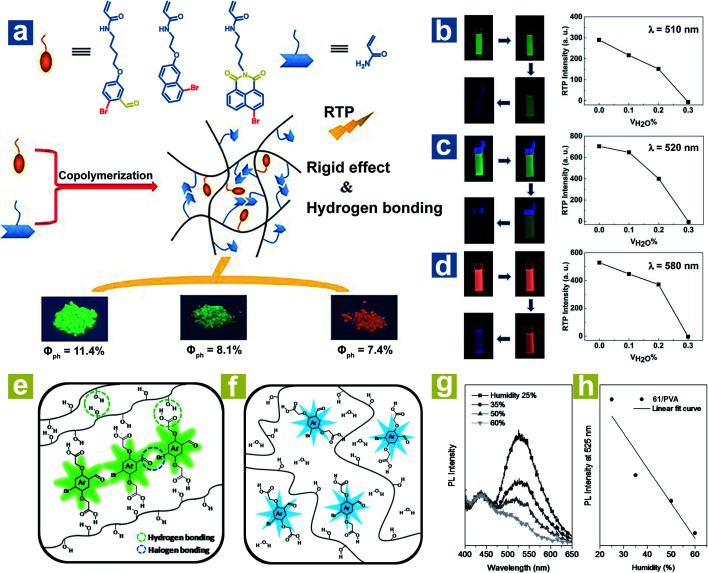
(a) Schematic illustration of OPL processes in polymers 58–60. Luminous photographs and OPL intensities of polymers (b) 58, (c) 59 and (d) 60 in different volume mixing ratios of DMF and H_2_O. Schematic illustration of phosphorescence processes in the polymer (64 in PVA) (e) with and (f) without water. (g) PL spectra (*λ*_ex_ = 365 nm) of the polymer film (1 wt% 64 in PVA) at various humidities and (h) the fitting curve of the PL intensity at 525 nm *versus* humidity. a–d, Reproduced from ref. 88 with permission from Copyright 2016 WILEY-VCH; e–h, Reproduced from ref. 89 with permission from Copyright 2014, WILEY-VCH.

To achieve a high-efficiency OPL emission, a rigid environment is usually adopted. The hydrogen bonds and halogen bonds in the rigid environment can be destroyed by water, leading to weak OPL emission. Tian and Ma *et al.* reported the halide-containing polymers 58–60 with OPL emission at 510, 520 and 580 nm.^88^ The hydrogen bonding between polymeric chains might immobilize the OPL phosphors (Fig. 8a), and the hydrogen bonding could be broken by water, leading to phosphorescence quenching. As shown in Fig. 8b–d, the phosphorescence emission gradually vanished with the increase of the proportion of water in the solvent. Kim *et al.* reported water-responsive polymer films doped with compound 64 in 80% hydrolysed PVA (PVA80) or 100% hydrolysed PVA (PVA100).^89^ The vibration of phosphors is restricted seriously by the strong H-bonds between the carboxylic acid periphery in 64 and the hydroxyl in PVA, and the intermolecular halogen bond Br⋯O between the phosphors (Fig. 8e), leading to small *k*_nr_ and efficient OPL emission. Also, the halogen bonds are conducive to the realization of high *k*_isc_. The water in air will be absorbed by the polymer film and break the H-bonds in PVA–PVA and PVA-64 (Fig. 8f). As the humidity increases, the phosphorescence intensity presents a linear decrease due to the ever-increasing *k*_nr_ (Fig. 8g and h). Furthermore, when water is dropped onto the film, halogen bonds between phosphors in the film will be also broken by the water, inducing the quenching of phosphorescence emission.

### 6.4 UV light sensing

**Fig. 9:**
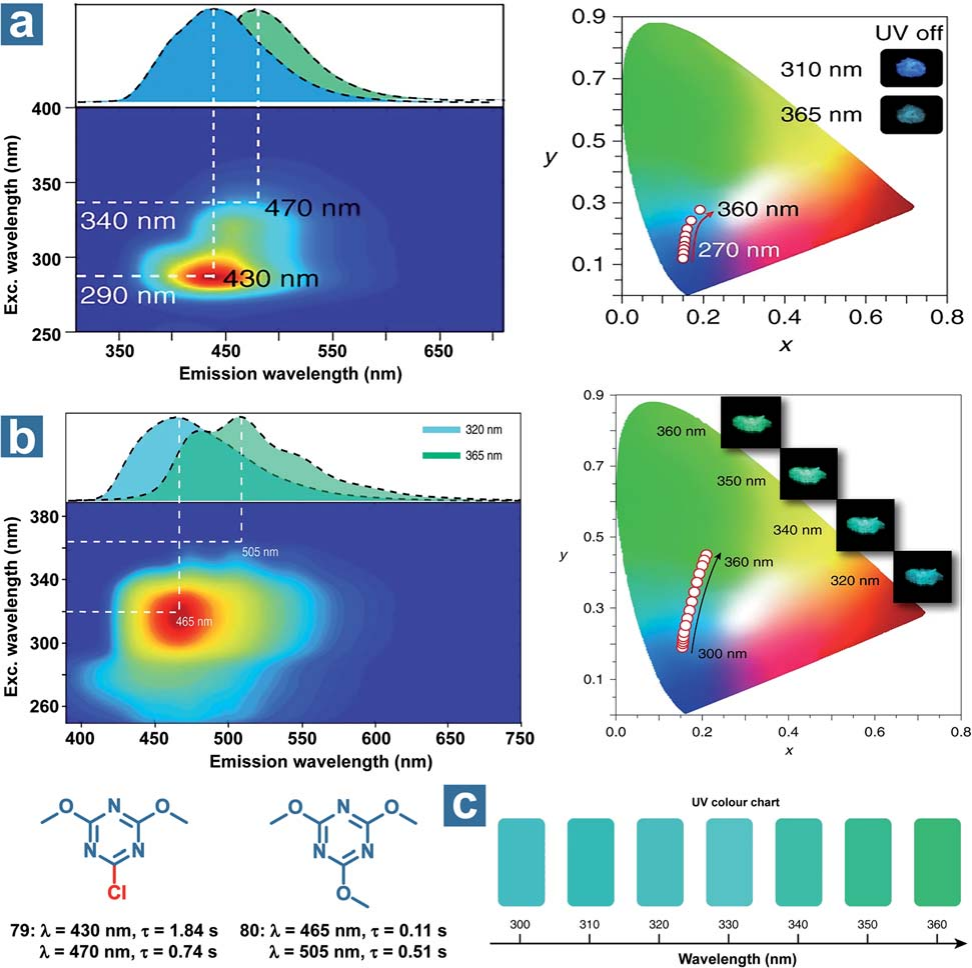
Excitation-phosphorescence mapping and OPL emission colors in CIE coordinate diagrams of (a) 79 and (b) 80. (c) UV colour chart showing the OPL colours of 80 crystalline powders at specific UV excitation wavelengths. Reproduced from ref. 108 with permission from Copyright 2019, Springer Nature.

The formation of multiple emitting centres in the OPL materials makes it possible for the tuneable emission colours at various excitation wavelengths, and thus they can be used for the visual detection of specific UV light. Huang and An *et al.* reported several triazine derivatives exhibiting tuneable OPL emission under UV excitation with distinct wavelengths.^108^ As shown in Fig. 9a and b, two different phosphorescence emission bands are observed from crystalline powder 79 and 80, which are regarded as the molecular phosphorescence and the ultra-long phosphorescence from aggregations. With a change in the excitation wavelength from 250 to 400 nm, the OPL emission of 79 exhibits an obvious bathochromic shift from sky-blue to blue-green along with a variation in the main emission peak from 430 to 470 nm. As the invisible UV excitation changes from 300 to 360 nm with an interval of 10 nm, distinguishable OPL colour can be captured by the naked eye (Fig. 9c), showing the ability of OPL materials in sensing specific UV wavelengths.

**Fig. 10:**
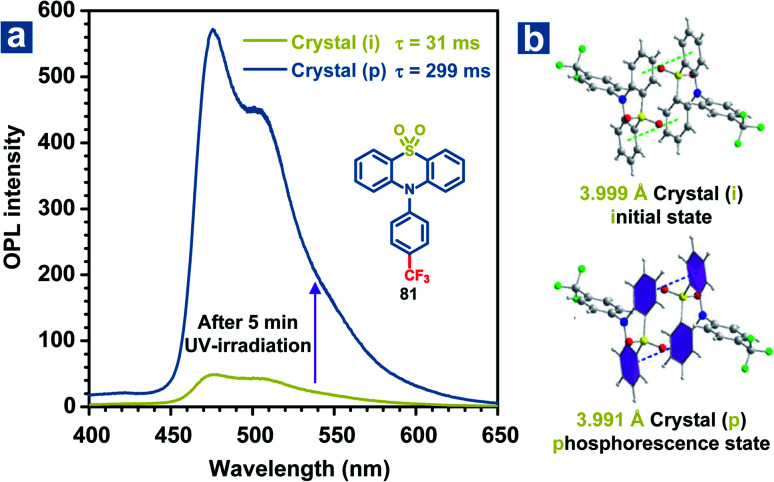
(a) Phosphorescence spectra of 81 crystals before (the green line) and after (the blue line) 365 nm UV irradiation for 5 minutes. (b) The crystal structures of 81 before and after UV irradiation. Crystal (i) refers to the crystal before UV irradiation, and crystal (p) refers to the crystal after UV irradiation for 5 minutes.

Besides, slight molecular motions in OPL materials or the altered molecular structures may be induced by UV irradiation, leading to momentous changes in the OPL emission intensity or lifetimes, which can be used for detecting UV light.^109^ Li and Pu *et al.* found that weak phosphorescence emission was observed from the crystal of 81, but it showed OPL emission with a lifetime of 0.3 s upon UV irradiation for 5 minutes (Fig. 10a).^51^ The crystal structures reveal that the distance between two adjacent phenyl rings in the coupled 81 changes from 3.999 Å to 3.991 Å after UV irradiation for 5 min (Fig. 10b), contributing to the enhanced π–π interactions and OPL emission with longer lifetimes. The photo-induced OPL properties will disappear without the UV irradiation for about 2 hours, showing the reversible change of crystal packing.

**Fig. 11:**
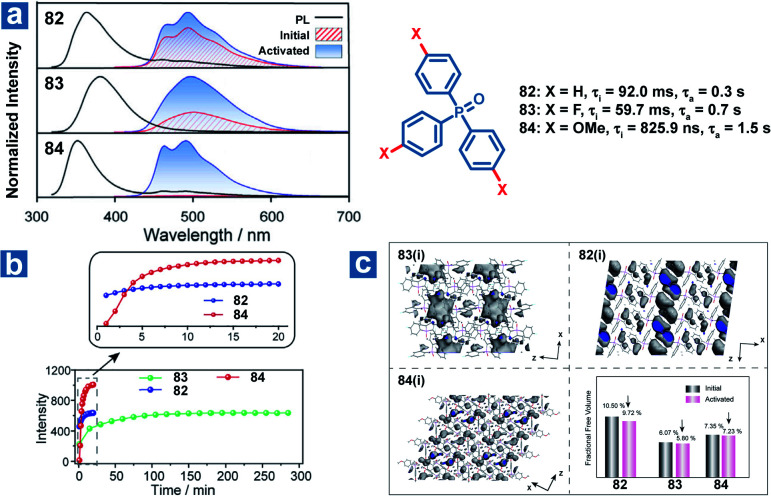
(a) Photoluminescence (black lines) and phosphorescence spectra of 82–84 before (red lines) and after (blue lines) photoactivation. (b) OPL intensity of 82–84 at 490 nm with increasing photoactivation time. (c) The free volume region (gray isosurface) and fractional free volume in 2 × 2 × 2 unit cells before and after photoactivation. Initial (i) refers to the state before photoactivation and activated (a) refers to the state after photoactivation.

Zhao and Huang *et al.* also reported controllable photoactivated OPL emission from triphenylphosphine oxide derivatives 82–84 (Fig. 11), and obviously increased OPL emission intensity and lifetimes can be observed after UV irradiation.^110^ Newly formed C⋯π (2.66–2.6 Å) interactions and closer intermolecular interactions are found in the crystals after UV irradiation. In addition, the reduced free volume distributions of crystals prove the more rigid molecular stacking upon prolonged photoirradiation. The introduction of the F element leads to stronger hydrogen bonding interactions (C–F⋯H and C–O⋯H) in compound 83 than in 82 and 84 in the initial state, contributing to longer photoactivation time for 83 (Fig. 11b).

### 6.5 Organic solvent sensing

**Fig. 12:**
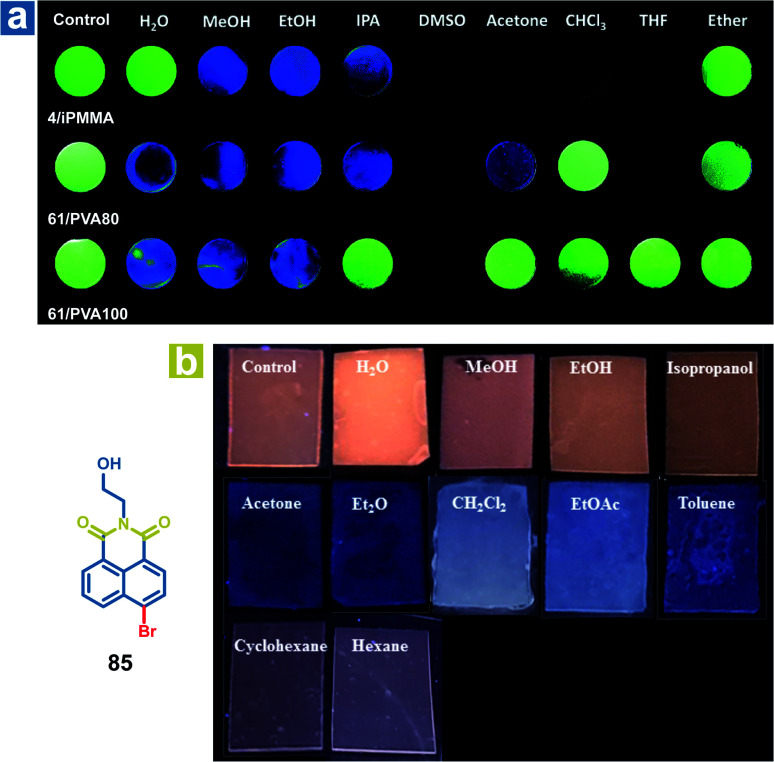
(a) Photographs of the 4/iPMMA, 64/PVA80, and 64/PVA100 electrospun polymer fibre mats before (control) and after exposure to various organic solvents under 365 nm UV light. (b) Photographs of 85/acrylate–VDC polymer films before (Ccntrol) and after contact with solvents under 365 nm UV light. (a) Reproduced from ref. 90 with permission from Copyright 2016, The Royal Society of Chemistry; (b) reproduced from ref. 111 with permission from Copyright 2020, American Chemical Society.

The mechanism of organic solvent sensing is similar to that of water sensing by OPL materials. The rigid environment of OPL phosphors is damaged upon exposure to organic solvents, leading to a change in the OPL emission properties. Kim *et al.* reported array-based selective detection of organic solvents using the polymers isotactic PMMA (iPMMA), 80% hydrolysed PVA (PVA80) or 100% hydrolysed PVA (PVA100), containing bromobenzaldehyde phosphors (4 or 64).^90^ Electrospun fibre mats are fabricated as the organic solvent sensing platform from the polymer doping systems, and the phosphorescence emission will disappear if the solvent dissolves the polymer matrix and breaks the halogen-bonded luminophore networks. As shown in Fig. 12a, iPMMA would be dissolved in polar organic solvents, leading to blue fluorescence or no emission from the polymer mat 4/iPMMA, but it can exhibit green phosphorescence emission after exposure to nonpolar ether. Liu and Chen *et al.* also reported polymer films to detect organic solvents, employing the acrylate-vinylidene chloride copolymers (acrylate–VDC) as the polymer matrix and *N*-hydroxyethyl 4-bromo-1,8-naphthalimide (85) as the OPL phosphor.^111^ As shown in Fig. 12b, the hydrophobic polymer matrix is immiscible with polar protic solvents, and the film keeps the OPL emission in contact with alcohol. Medium polar solvents such as acetone, diethyl ether, dichloromethane and ethyl acetate will swell the film and increase the free volume of the film, leading to the increasing mobility of phosphors and quenching of OPL emission. The nonpolar solvents may swell the film slightly due to their hydrophobicity, leading to weak OPL emission upon exposure to cyclohexane and hexane. To sum up, the response of the polymer films to organic solvents or water depends on the interaction between the polymer matrix and the solvents. Then, the changed environment of the phosphor results in various OPL emission properties.

**Fig. 13:**
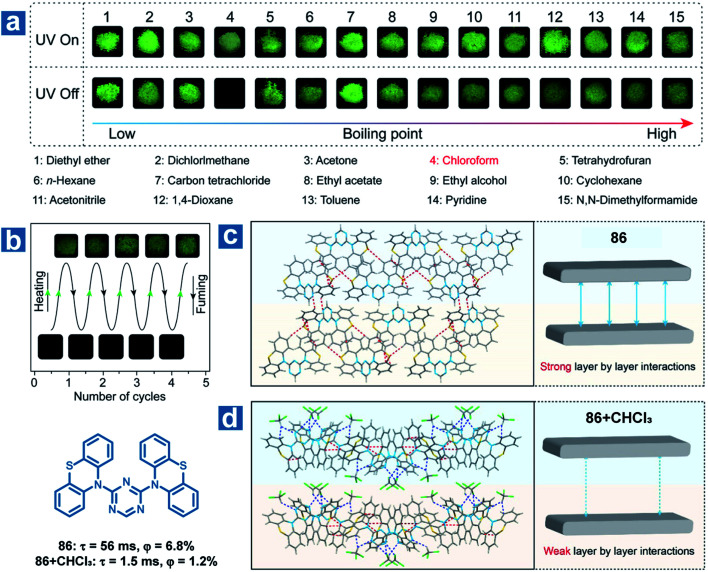
(a) Photographs of 86 powder fumed with various organic solvents for 3 h with a 365 nm UV lamp on and off. (b) Photographs of reversible OPL emission of 86 by fuming with chloroform and heating. Intermolecular stacking of 86 crystals (c) without and (d) with chloroform molecules. Reproduced from ref. 112 with permission from Copyright 2018, American Chemical Society.

Furthermore, the solvent molecule can influence the crystal structures of the OPL phosphors directly accompanied by the variation in OPL emission. Huang and An *et al.* found that the OPL emission of the crystal 86 can be quenched by chloroform and the material can be used as a visible and selective chloroform indicator (Fig. 13).^112^ The insertion of chloroform molecules into the crystals leads to looser molecular packing, with fewer intermolecular interactions and weaker layer-by-layer interactions (Fig. 13d). Thus, larger *k*^T^_nr_, lower RTP efficiency and shorter lifetimes are found in the crystals containing chloroform molecules than the single-component crystals. As exhibited in Fig. 13, 86 shows a high selectivity for chloroform detection, and there is no change in the OPL properties of the material after fuming with other 14 types of volatile organic solvents. In addition, the compound shows high repeatability and a low chloroform detection limit of 5 ppm.

**Fig. 14:**
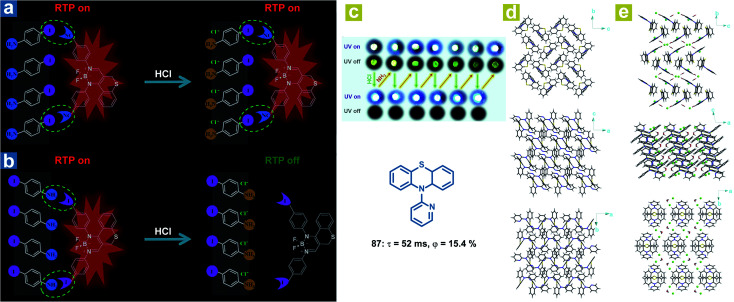
Schematic illustration the different HCl-responsive behaviors of doped crystals (a) 76/75 and (b) 77/75. (c) Photographs of reversible OPL emission of 87 by fuming with HCl and NH_3_. Molecular packing of 87 crystals (d) without and (e) with chloroform molecules. a, Reproduced from ref. 46 with permission from Copyright 2018, Wiley-VCH.

Interestingly, in addition to the organic chloroform molecules, Fu *et al.* reported hydrochloric acid (HCl) induced phosphorescence quenching for the destroying of halogen bonds (Fig. 14a and b).^46^ The halogen bonds CN⋯I–Ph–NH_2_ and I⋯NH_2_–Ph–I are identified in the doped crystals with 4-iodoaniline (I–Ph–H_2_, 75) as the host crystalline matrix and the chromophores (76 or 77) as the guest molecules. After treatment with HCl, the formation of I–Ph–NH_2_·HCl destroys the original halogen bonds, leading to loose restrictions on the chromophore and the weakened OPL emission. Halogen bonding CN⋯I in the doped crystal 76/75 remains after the formation of I–Ph–NH_2_·HCl, leading to no change in the red OPL emission. Li and Fang *et al.* also found that OPL emission can be quenched by the HCl vapor (Fig. 14c).^113^ A completely different molecular aggregation of the compound 87 is formed after incubation with hydrochloric acid solution, and the HCl and H_2_O molecules are also involved in the molecular arrangement. The intermolecular hydrogen bonds (C–H⋯Cl, and O–H⋯Cl) reduced the intermolecular electron interactions between the adjacent two molecules (Fig. 14d and e), contributing to the restricted intermolecular charge transfer, much weakened ISC process, and quenched OPL emission. Interestingly, the OPL properties can be resumed after fuming with ammonia vapor.

## 7. Conclusion and perspectives

In summary, this review highlights the current research progress of the development and sensing application of halide-containing OPL materials, and focuses on the advantages of these materials for sensing applications. Changes in the photophysical properties, like emission lifetimes and intensity, will be observed in halide-containing OPL materials under different environmental conditions. These visible optical changes are used as the indicating signals to sense temperature, oxygen, H_2_O, UV light or organic solvents. Based on the photophysical process of OPL emission, three environmental sensing mechanisms of OPL materials are concluded. (1) Endothermic nonradiative transition and the RISC process induce notable changes in the lifetimes and intensity of the phosphorescence and TADF emission at different temperatures, coinciding with various OPL colours. (2) Spin-allowed energy transfer from triplet states of OPL materials to oxygen makes oxygen an effective quencher of OPL emission. Also, the long emission lifetime makes OPL materials more sensitive to oxygen according to the Stern–Volmer kinetic relationships. (3) The decrease in the OPL emission intensity and lifetime can be induced by the breaking of the rigid environment of the phosphors or the changed molecular stacking, which may be generated in OPL materials upon exposure to specific species.

**Scheme 8:**
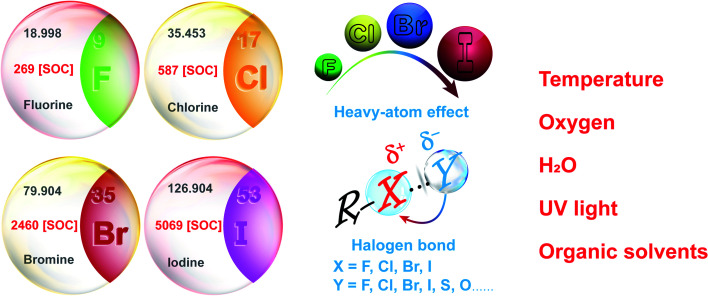
Influence of halogen elements on the OPL materials and their sensing applications.

In addition, the design and classification of halide-containing OPL material systems have been summarized, and halide elements exhibit significant positive effects on the OPL emission and sensing applications (Scheme 8). (1) F, Cl, Br, and I present different heavy-atom effects, which will facilitate the OPL emission greatly, and the atomic spin–orbit coupling constants increase remarkably from 269 cm^−1^ for F to 5069 cm^−1^ for I.^43^ Thus, larger *k*_ISC_ and *k*^T^_r_ can be usually realized in the iodine-containing materials, exhibiting higher OPL efficiency but relatively shorter emission lifetimes than fluorine- or chlorine-containing materials. (2) Both the aromatic halogen atoms and halogen ions can form the halogen bonds R–X⋯Y (Scheme 8), and the bonding interaction increases gradually when X in R–X⋯Y changes from F, Cl to Br or I elements.^47,114^ The halogen bonding contributes to serious restrictions on the OPL luminophores and significant suppression of nonradiative transitions, leading to long emission lifetimes. (3) Enhanced heavy-atom effects may be induced by the halogen bonding to boost the OPL emission. Because electrons on the Y atoms in the interaction R–X⋯Y are delocalized partially to the halogen atoms X, it will promote the electron spin–orbit interaction of X. (4) The electron-withdrawing characteristics of the aromatic halogen atoms lead to a decrease in the π-electron density and relieves the π–π repulsion between adjacent aromatic rings, which is beneficial for the construction of a rigid face-to-face stacking environment.^51^ Also, the halogen substituents have proved to strengthen the edge-to-face interaction between the aromatic derivatives.^50^ As shown above, the introduction of halogen elements and the formation of halogen bonds will result in highly efficient OPL emission. Conversely, the destroying of original halogen bonding networks or the rigid environment by the external stimulation will induce the quenching of OPL emission, which can be utilized for the environmental sensing. Also, OPL materials containing different halogen elements exhibit various emission lifetimes and efficiency, which will satisfy different application scenarios.^10^

In spite of the advantages and current achievements of the halide-containing OPL materials, there are still some challenges and opportunities in the research of OPL sensors. (1) Inherent properties of triplet excitons, which are sensitive to oxygen and temperature, make the OPL materials able to sense oxygen and temperature. For these kinds of OPL sensors, higher quantum yields and longer lifetimes should be realized simultaneously in the future to improve the sensitivity and detection limit. (2) Most of the halide-containing OPL materials require a rigid environment (crystalline or polymer matrix), limiting the sensing application in aqueous solution, such as the pH or ion detection. It is urgent to develop novel halide-containing nanoparticles or nanocrystals exhibiting OPL emission in solution, which is essential for biosensing.^115,116^ (3) Chemical changes will lead to various emission properties, which is common for the traditional luminescent sensors,^117–119^ but this sensing mechanism is only observed in a few OPL materials. Reactive response is conducive to increase the selectivity and accuracy of OPL sensors, and the specific response of OPL materials to chemical species is also an area worthy of exploring.

With the enrichment of materials, the refinement of characterization and the expansion of applications, the research on halide-containing OPL materials will continue to develop rapidly. Furthermore, the related theories of environmental sensing can also be developed. We hope this review can provide some help for future research directions and contribute to the further development of OPL materials for environmental sensing.

## Author contributions

Zhao and Liu conceived this review, and Zhao, Li and Wang conducted the literature review. Li and Wang wrote the manuscript, and all authors contributed to the revision of the manuscript.

## Conflicts of interest

There are no conflicts to declare.

## Supplementary Material
